# Subcortical hyperintensity volumetrics in Alzheimer’s disease and normal elderly in the Sunnybrook Dementia Study: correlations with atrophy, executive function, mental processing speed, and verbal memory

**DOI:** 10.1186/alzrt279

**Published:** 2014-08-11

**Authors:** Joel Ramirez, Alicia A McNeely, Christopher JM Scott, Donald T Stuss, Sandra E Black

**Affiliations:** 1 LC Campbell Cognitive Neurology Research Unit, 2075 Bayview Avenue, Room A4 21, Toronto, ON M4N 3M5, Canada; 2 Heart & Stroke Foundation Canadian Partnership for Stroke Recovery, Toronto, ON, Canada; 3 Sunnybrook Health Sciences Centre, Brain Sciences Research Program, Sunnybrook Research Institute, Toronto, ON, Canada; 4 Faculty of Medicine, Institute of Medical Science, University of Toronto, Toronto, ON, Canada; 5 Rotman Research Institute, Baycrest, Toronto, ON, Canada; 6 Ontario Brain Institute, Toronto, ON, Canada

## Abstract

**Introduction:**

Subcortical hyperintensities (SHs) are radiological entities commonly observed on magnetic resonance imaging (MRI) of patients with Alzheimer’s disease (AD) and normal elderly controls. Although the presence of SH is believed to indicate some form of subcortical vasculopathy, pathological heterogeneity, methodological differences, and the contribution of brain atrophy associated with AD pathology have yielded inconsistent results in the literature.

**Methods:**

Using the Lesion Explorer (LE) MRI processing pipeline for SH quantification and brain atrophy, this study examined SH volumes of interest and cognitive function in a sample of patients with AD (n = 265) and normal elderly controls (n = 100) from the Sunnybrook Dementia Study.

**Results:**

Compared with healthy controls, patients with AD were found to have less gray matter, less white matter, and more sulcal and ventricular cerebrospinal fluid (all significant, *P* <0.0001). Additionally, patients with AD had greater volumes of whole-brain SH (*P* <0.01), periventricular SH (pvSH) (*P* <0.01), deep white SH (dwSH) (*P* <0.05), and lacunar lesions (*P* <0.0001). In patients with AD, regression analyses revealed a significant association between global atrophy and pvSH (*P* = 0.02) and ventricular atrophy with whole-brain SH (*P* <0.0001). Regional volumes of interest revealed significant correlations with medial middle frontal SH volume and executive function (*P* <0.001) in normal controls but not in patients with AD, global pvSH volume and mental processing speed (*P* <0.01) in patients with AD, and left temporal SH volume and memory (*P* <0.01) in patients with AD.

**Conclusions:**

These brain-behavior relationships and correlations with brain atrophy suggest that subtle, yet measurable, signs of small vessel disease may have potential clinical relevance as targets for treatment in Alzheimer’s dementia.

## Introduction

Subcortical hyperintensities (SHs) are commonly observed radiological entities on T2-weighted (T2), proton density (PD), and fluid attenuated inversion recovery (FLAIR) magnetic resonance images (MRIs) of the aging brain [1,2]. Often referred to as leukoaraiosis, these diffuse white matter (WM) abnormalities appear as hyperintense bright spots on PD/T2 MRIs and are believed to reflect some form of small vessel disease [3-5]. Although the pathological origins of SH and their contribution to the expression of dementia remain controversial, recent studies have shown SH to be associated with cognitive function, gait disturbances, and mental processing speed [6-13].

SH can be subclassified as periventricular (pvSH) and deep white (dwSH) [14-16]. Additionally, cystic lacunar infarcts can be measured if T1-weighted (T1) acquisitions are obtained [17,18]. Standard brain tissue segmentation algorithms typically include MRI-derived volumetric estimates for gray matter (GM), WM, sulcal cerebrospinal fluid (sCSF), and ventricular CSF (vCSF). However, since T1-based segmentations may result in misclassified tissue volumetrics due to the relative intensity of SH on T1 [17], an additional PD/T2/FLAIR-based SH segmentation is recommended to correct for this error and account for the possible contribution of ischemic vascular injury, particularly with studies examining aging and dementia [19].

The purpose of the present study is to better understand the complex relationships between MRI measures of small vessel disease, atrophy, and cognition in patients with sporadic Alzheimer’s disease (AD) and cognitively normal elderly controls (NCs). More specifically, this study used MRI-derived (T1/T2/PD) volumetrics to determine whether regional SH volumes of interest (VOIs) were differentially correlated with atrophy and performance on tasks probing executive function, mental processing speed, and memory.

## Materials and methods

### Participants

MRI scans were obtained on patients with AD (AD: n = 265) and cognitively normal elderly volunteers (NC: n = 100) enrolled in the Sunnybrook Dementia Study (ClinicalTrials.gov: NCT01800214), which is a large ongoing longitudinal study conducted in the LC Campbell Cognitive Neurology Research Unit and the Heart & Stroke Foundation Canadian Partnership for Stroke Recovery, at Sunnybrook Health Sciences Centre in Toronto, Canada.

Patients with AD were slightly older (AD = 72.8 ± 9.0 versus NC = 69.5 ± 8.0, *P* <0.001) and less educated (AD = 13.8 ± 3.8 versus NC = 15.5 ± 3.0, *P* <0.001). The presence/non-presence of SH was not an exclusionary criterion for this study, and both groups showed a non-normal distribution of SH volumes. Participants were excluded if they had signs of Parkinson’s disease or neurological diseases other than dementia, history of significant head trauma, psychotic disorders unrelated to dementia, psychoactive substance abuse, major depression, and other clinically significant pathology such as overt stroke, tumors, or normal pressure hydrocephalus. Patients with AD met National Institute of Neurological and Communicative Disorders and Stroke (NINCDS) Alzheimer’s Disease and Related Disorders Association (ADRDA) criteria for probable or possible AD [20] and *Diagnostic and Statistical Manual of Mental Disorders* (4th ed.) [21] criteria for dementia. All patients received a standardized comprehensive clinical evaluation. NC participants were community-dwelling with no subjective or objective cognitive impairment and no history of significant psychiatric or neurological diseases. All NC met strict criteria, including pre-screening followed by a battery of neuropsychological testing with performance threshold requirements for consideration. The study protocol was approved by the Sunnybrook Research Ethics Board (REB PIN no. 009-1998), and written informed consent was obtained from all participants or their caregivers/decision-makers or both.

### Neuropsychological tests

Neuropsychological testing was performed within 12 weeks of MRI acquisition and administered according to standard protocols. Executive function, mental processing speed, and memory domain tests were selected because these domains have been most consistently associated with SH in the previous literature. (See Table 1 for specific measures used.) Executive function was assessed by using performance scores obtained from the F-A-S phonemic verbal fluency task [22] and the Wisconsin Card Sorting Test [23]. The time to complete the Trails A trail-making test was used to assess speed of mental processing [24]. The California Verbal Learning Test [25], Wechsler Memory Scale-Revised immediate Visual Reproduction [26], and the Dementia Rating Scale memory subscore [27] were used to assess memory. Composite scores were generated by using normalized mean z-scores and normal elderly control population data from the Sunnybrook Dementia Study.

**Table 1:** Summary of cognitive domains and measures used to generate composite scores

**Domain**	**Test**	**Measure**
**Executive**	Verbal Fluency ‘FAS’ Test	Total words correct
	Wisconsin Card Sorting Test	Total correct
	Wisconsin Card Sorting Test	Perseverative errors to previous response
	Wisconsin Card Sorting Test	Perseverative errors to previous category
**Mental processing speed**	Trail Making Test (part A)	Time to complete (seconds)
**Memory**	California Verbal Learning Test	Total correct at acquisition
	Wechsler Memory Scale Revised Visual Reproduction	Immediate recall
	Dementia Rating Scale	Memory subscore

### MRI acquisition protocols

All brain imaging data were obtained on a 1.5 Tesla GE Signa (Milwaukee, WI, USA) system in compliance with consensus recommendations for studies examining vascular cognitive impairment [19]. Three image sets were used: a T1-weighted—axial three-dimensional (3D) Spoiled Gradient Recalled Echo (SPGR): 5 ms echo time (TE), 35 ms repetition time (TR), 1 number of excitations (NEX), 35° flip angle, 22 × 16.5 cm (FOV), 0.859 × 0.859 mm in-plane resolution, 1.2 to 1.4 mm slice thickness depending on head size—and an interleaved PD and T2 (interleaved axial dual-echo spin echo: TEs of 30 and 80 ms, 3 s TR, 0.5 NEX, 20 × 20 cm FOV, 0.781 × 0.781 mm in-plane resolution, 3 mm slice thickness).

### MRI processing

Full methodological details and reliability results of the image processing pipeline are previously published [18,28]. In brief, the Lesion Explorer (LE) processing pipeline (available for download at http://sabre.brainlab.ca), a tri-feature (T1/PD/T2) segmentation and parcellation procedure, was applied to obtain regionalized and whole-brain volumetrics for GM, WM, sCSF, vCSF, periventricular SH (pvSH), deep white SH (dwSH), and cystic fluid filled lacunar-like infarcts (lacunes). The Brain-Sizer component of LE effectively removed skull and other non-brain structures to obtain a reliable measure of supra-tentorial total intracranial volume (ST-TIV), a process which included a measure of subarachnoid CSF immediately below the dura mater. A robust T1-based basic tissue (GM, WM, sCSF) segmentation, which fits localized voxel intensities to four Gaussian curves, was performed [29]. The Semi-Automatic Brain Region Extraction (SABRE) component of LE parcellated the brain into 26 standardized VOIs described in a previous publication [30]. The third component of LE segmented SH from PD/T2, parcellated each volume into the 26 SABRE VOIs, separated SH volumes into pvSH and dwSH by using a 3D connectivity algorithm, and further segmented lacunar-like infarcts within each SH volume by using information from the initial T1 segmentation. The final output provided a comprehensive volumetric profile for each individual that included volumetrics within 26 SABRE VOIs for GM, WM, sCSF, vCSF, pvSH, dwSH, and lacunes.

Terminology and definitions were in compliance with the international neuroimaging standards recently recommended by the STandards for ReportIng Vascular changes on nEuroimaging (STRIVE) [2].

### Statistical analyses

Although mean raw volumetric data are presented for transparency purposes and volumetric comparisons across studies (Table 2), all statistical analyses were performed on normalized values following a previously published standard procedure [31] and subsequently converted to z-scores (with log transformation when required). Between-group volumetric differences (AD versus NC) were compared by using analysis of covariance (ANCOVA). Cohen’s *d* effect sizes were calculated for significant between-group comparison results by using the pooled standard deviation method on corrected data (note: volumetric tables show raw data). A standardized *a priori* VOI-based analysis approach was used to test the SABRE VOIs. In brief, step-wise modeling for exploratory analyses combined the SABRE VOIs for each of the four major lobes (frontal, parietal, temporal, and occipital) which yielded reasonable associations for our VOIs. After localizing the correlations to a particular lobe, subregional parcellations within each SABRE subregion were entered into the *a priori* subparcels into each regression model, which was limited to a maximum of six VOIs per model. Within-group SH VOI correlations with cognitive performance scores were assessed by using linear regression with backwards elimination of non-significant variables. All analyses accounted for age, sex, years of education, and brain parenchymal fraction (BPF), where BPF was defined as a measure of whole-brain atrophy, calculated by dividing the total parenchymal volume (GM + WM) by the ST-TIV. After removal of the cerebellum and subtentorial structures, ST-TIV was calculated for each individual and included all parenchyma (GM + WM), SH and lacunar volumes, sulcal and ventricular CSF, and all CSF immediately below the dura mater to provide an accurate measure of the intracranial cavity. In addition to BPF, head size-corrected vCSF volumes (vCSF/ST-TIV) were analyzed as another commonly used imaging biomarker of brain atrophy [32-34]. Age, sex, years of education, and scores from the Mini-Mental State Exam (MMSE) [35] were entered as covariates to account for differences in disease severity.

**Table 2:** Participant demographics and volumetric imaging statistics

	**AD**	**NC**	***P *** **value**	**Cohen’s *d *** **effect size**
**Demographics**				
	Number	265	100	-	-
	Age, years	72.8 (9.0)	69.5 (8.0)	<0.001	0.38
	Sex, number (percentage) female	152 (57)	55 (55)	-	-
	Education, years	13.8 (3.8)	15.5 (3.0)	<0.001	0.47
	MMSE/30^a^	23.2 (4.5)	29.0 (1.1)	<0.05	1.49
**Basic tissue volumetrics**^**b**^					
	ST-TIV	1,211.8 (140.1)	1,227.7 (112.5)	n.s.	-
	BPF%	73.1 (4.6)	79.0 (3.7)	<0.0001	1.35
	GM	509.9 (55.7)	560.8 (45.0)	<0.0001	1.35
	WM	363.6 (55.1)	403.1 (52.3)	<0.0001	0.92
	sCSF	274.8 (62.5)	224.2 (48.1)	<0.0001	1.24
	vCSF	52.9 (27.1)	34.1 (16.2)	<0.0001	1.01
**SH volumetrics**					
	SH, median (IQR)	5.4 (11.0)	2.5 (3.3)	<0.01	0.54
	pvSH, median (IQR)	4.5 (9.9)	1.8 (3.0)	<0.01	0.51
	dwSH, median (IQR)	0.6 (1.1)	0.3 (0.6)	<0.05	0.38
	Lacunar, mm^3^, median (IQR)	32.8 (155.9)	10.3 (45.0)	<0.0001	0.58

Values reported are mean (standard deviation) unless otherwise specified. ^a^Available in 97 cognitively normal elderly controls (NC) and 258 Alzheimer’s disease (AD) subjects. ^b^All volumes expressed in cubic centimeters unless otherwise indicated. BPF, brain parenchymal fraction; dwSH, deep white subcortical hyperintensity; GM, gray matter; IQR, interquartile range; MMSE, Mini-Mental State Exam; n.s., not significant; pvSH, periventricular subcortical hyperintensity; sCSF, sulcal cerebrospinal fluid; SH, subcortical hyperintensity; ST-TIV, supratentorial total intracranial volume; vCSF, ventricular cerebrospinal fluid; WM, white matter.

## Results

Demographic and volumetric results are presented in Table 2. ST-TIV was not significantly different and was comparable to those reported in the literature (*P* = 0.20, n.s.). Compared with NC, patients with AD were found to have less overall brain matter (smaller BPF%), less GM, less WM, and more sCSF and vCSF (all significant, *P* <0.0001), and relatively large effect sizes were demonstrated for overall brain atrophy and all basic tissue type comparisons (BPF%: *d* = 1.35, GM: *d* = 1.35, WM: *d* = 0.92, sCSF: *d* = 1.24, vCSF: *d* = 1.01). In addition, patients with AD had greater volumes of whole-brain SH (p <0.01), pvSH (*P* <0.01), dwSH (*P* <0.05), and lacunes (*P* <0.0001), and a medium effect size was demonstrated for whole-brain SH, pvSH, and lacunar volumes (SH: *d* = 0.54, pvSH: *d* = 0.51, lacunes: *d* = 0.58), and a smaller effect size for dwSH (*d* = 0.38).

**Figure 1:**
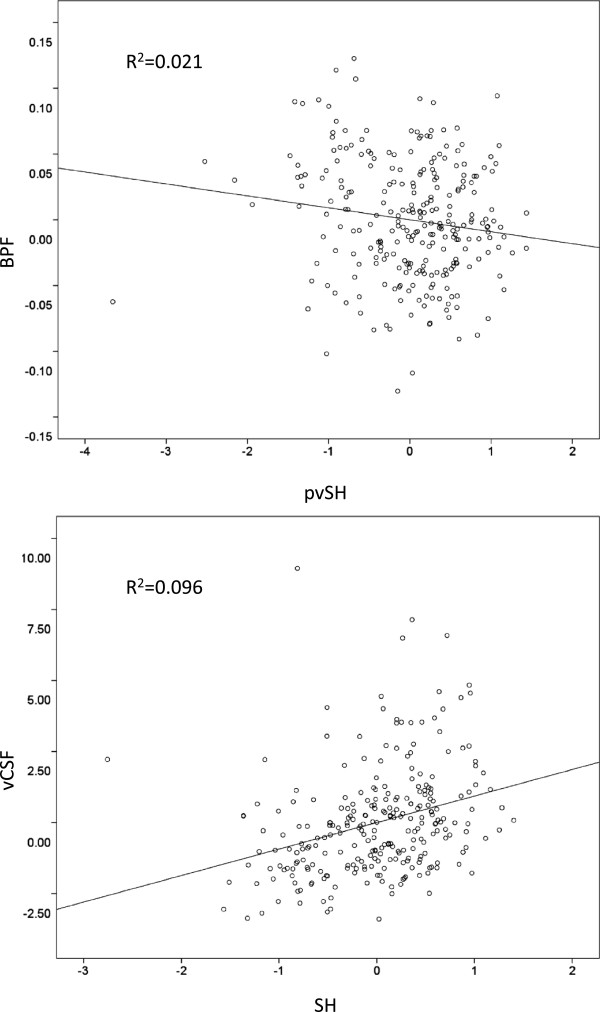
Partial regression plots illustrating the relationships in Alzheimer’s disease patients between atrophy measured by brain parenchymal fraction (BPF) and periventricular subcortical hyperintensity (pvSH) volume (top), and between atrophy measured by ventricular cerebrospinal fluid (vCSF) volume and subcortical hyperintensity (SH) volume (bottom).

**Figure 2:**
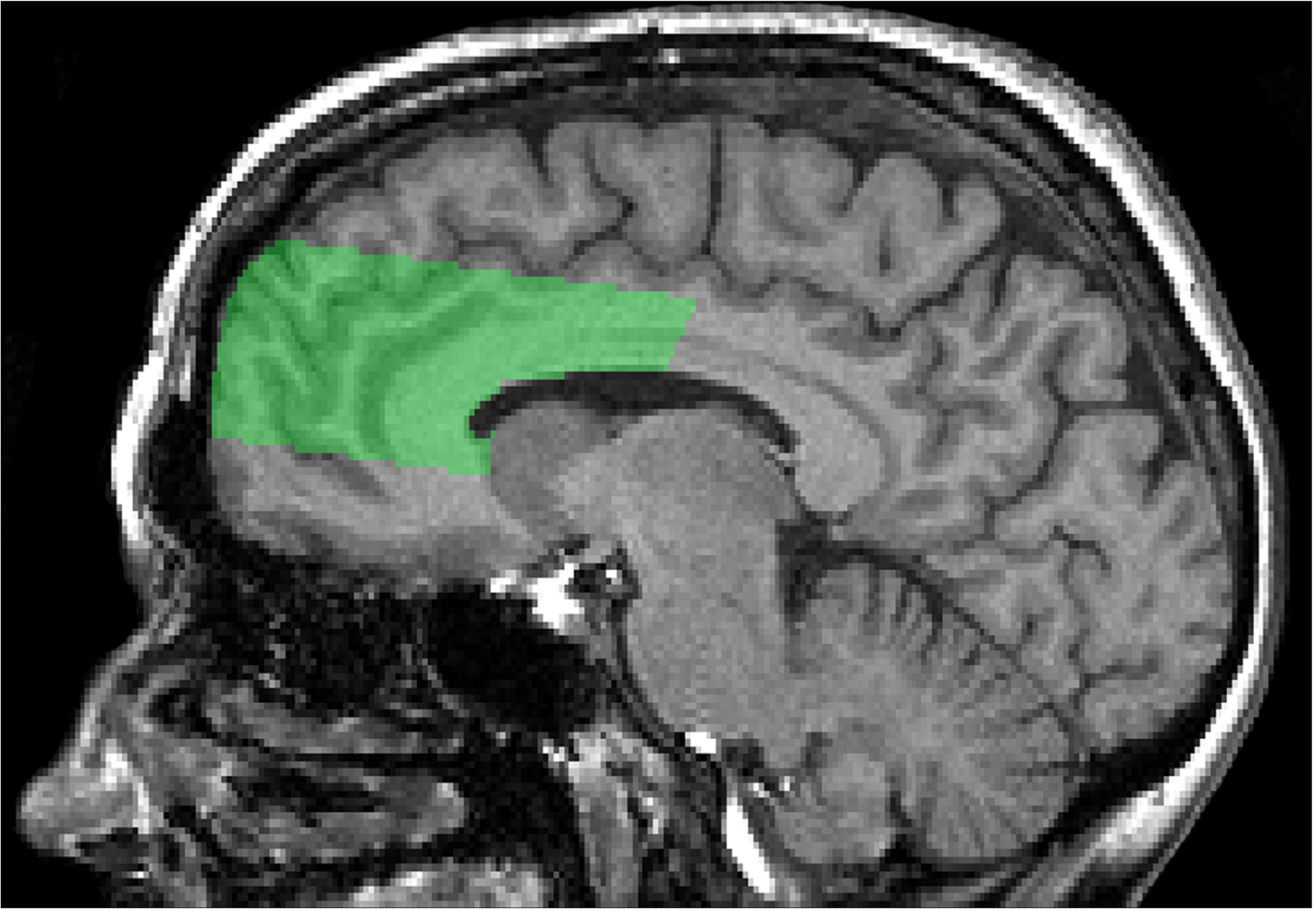
**Regional Semi-Automatic Brain Region Extraction (SABRE) parcellation.** Medial middle frontal is displayed in green on sagittal T1.

**Figure 3:**
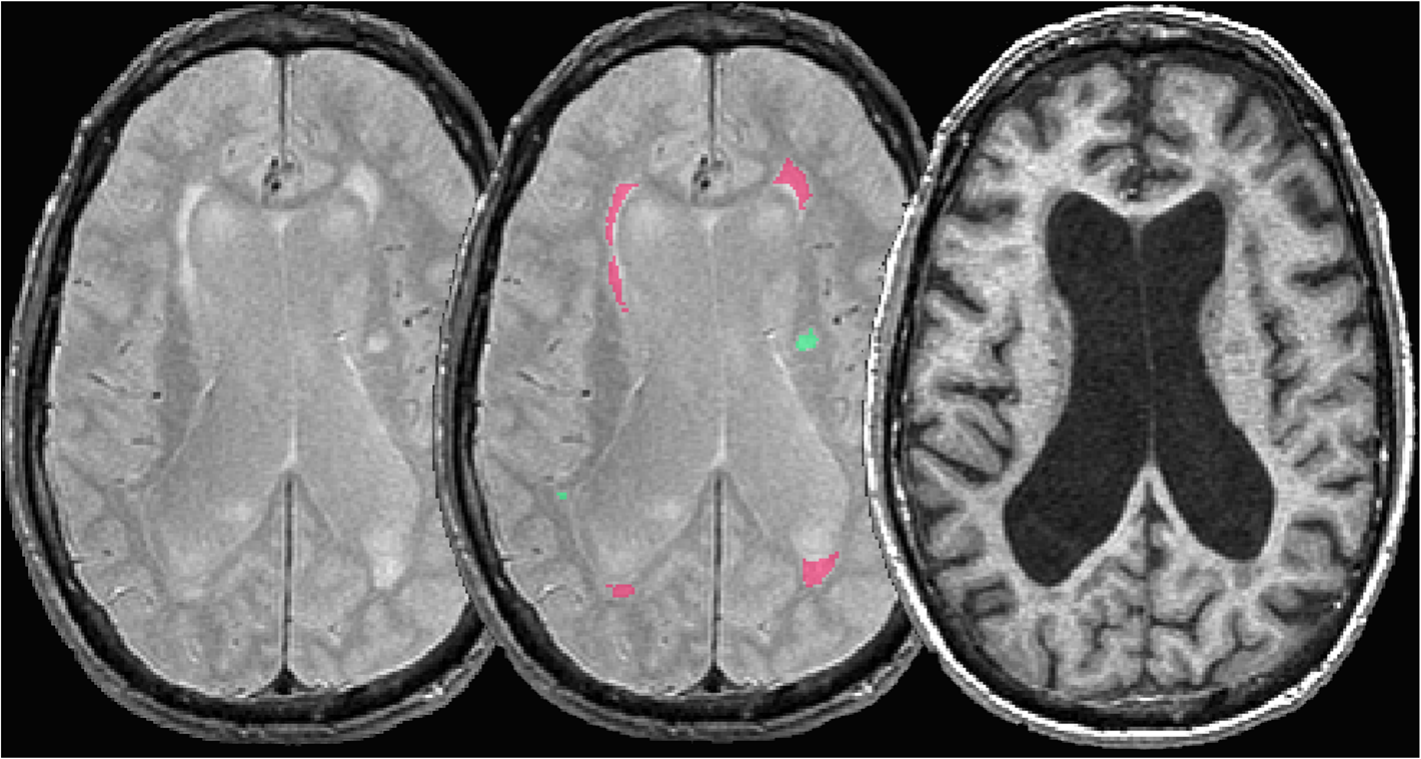
Segmentation of periventricular subcortical hyperintensity (pink) and deep white subcortical hyperintensity (green) overlayed on coregistered proton density (center) and T1-weighted (right) magnetic resonance imaging.

**Figure 4:**
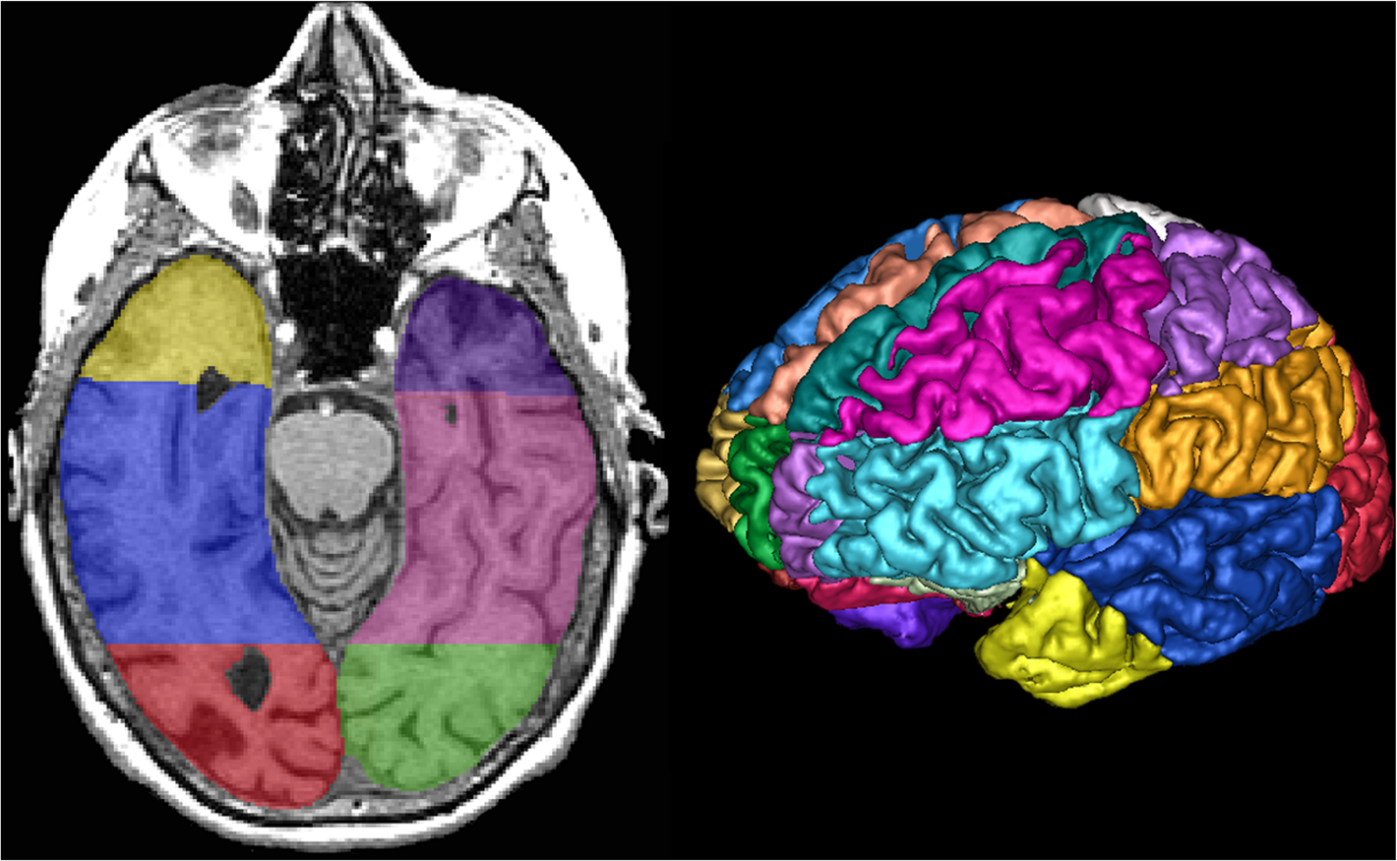
**Regional Semi-Automatic Brain Region Extraction (SABRE) parcellation.** Posterior temporal lobe is displayed in blue on axial T1 (left) and three-dimensional eroded T1 (right).

Significant within-group correlations with atrophy and cognitive performance scores are summarized in Table 3. In AD, regression analyses revealed a significant correlation with atrophy measured by BPF and pvSH volume (β = −0.14, R^2^ = 0.02, *P* = 0.02) and atrophy measured by vCSF and total SH (β = 0.31, R^2^ = 0.10, *P* <0.0001) (Figure 1). Medial middle frontal (MMF) SH volumes were significantly correlated with executive function in NC (β = −0.24, R^2^ = 0.07, *P* = 0.01) but not in AD (Figure 2). Mental processing speed was significantly correlated with pvSH volume in AD (β = 0.17, R^2^ = 0.03, *P* = 0.01) but not in NC (Figure 3). Additionally, memory was significantly correlated with left temporal SH volumes in AD only (β = −0.13, R^2^ = 0.02, *P* <0.05) (Figure 4). Overall, patients with AD demonstrated highly significant correlations with BPF in all cognitive domains examined (Executive: β = 0.26, R^2^ = 0.07, *P* <0.0001; Mental Processing Speed: β = −0.31, R^2^ = 0.08, *P* <0.0001; Memory: β = 0.30, R^2^ = 0.09, *P* <0.0001).

**Table 3:** Summary of significant regression β coefficients with subcortical hyperintensity volumetrics and cognitive domains

			**β coefficient**	**R**^**2**^	***P *** **value**
**Atrophy, measured by BPF**					
	AD (n = 265)				
		pvSH	−0.14	0.02	0.02
		Education, years	−0.16	0.03	0.009
		MMSE	0.12	0.01	0.06
	NC (n = 100)				
		Age	−0.64	0.42	<0.0001
		Sex	0.16	0.04	<0.05
**Atrophy, measured by vCSF**					
	AD (n = 265)				
		Total SH	0.31	0.10	<0.0001
		Education, years	0.14	0.02	0.02
		Sex	−0.12	0.02	0.04
	NC (n = 100)				
		Age	0.49	0.24	<0.0001
**Executive**					
	AD (n = 223)				
		BPF	0.26	0.07	<0.0001
	NC (n = 94)				
		Medial middle frontal SH	−0.24	0.07	0.01
		Education, years	0.23	0.06	<0.05
		BPF	0.31	0.10	<0.01
**Mental processing speed**					
	AD (n = 222)				
		pvSH	0.17	0.03	0.01
		Age	−0.33	0.08	<0.0001
		BPF	−0.31	0.08	<0.0001
	NC (n = 88)				
		Age	0.24	0.06	<0.05
**Memory**					
	AD (n = 236)				
		Left temporal SH	−0.13	0.02	<0.05
		Education, years	0.22	0.05	0.001
		BPF	0.30	0.09	<0.0001
	NC (n = 95)				
		Education, years	0.25	0.08	<0.01
		Age	−0.40	0.18	<0.0001

AD, patients with Alzheimer’s disease; BPF, brain parenchymal fraction; MMSE, Mini-Mental State Exam; NC, cognitively normal elderly controls; pvSH, periventricular subcortical hyperintensity; SH, subcortical hyperintensity; vCSF, ventricular cerebrospinal fluid.

## Discussion

Despite numerous clinically based research studies, the clinical relevance of SH remains to be fully elucidated. The most well-documented risk factors for presence of SH are aging, hypertension, and other cerebrovascular risk factors [36-39]. SHs have also been associated with cognitive decline, particularly speed of information processing and executive functions [40-43], as well as physical disability, particularly gait disorders and poor motor dexterity [12,44-47]. In neuropsychological studies, correlations with poor attention and reduced speed of mental processing have been consistent across many series [36,48,49].

In patients with AD, SHs have been previously correlated with MMSE scores and Clinical Dementia Rating scores, even after adjusting for potentially compounding variables such as age, sex, and cardiovascular risk factors [50]. Additionally, periventricular WM changes have been reported in 48% to 100% of AD cases [51,52]. However, when simultaneously considered with measures of GM and WM atrophy, SHs often explain only a small proportion of the variance, usually in relation to executive function measures [13,53,54].

The main results of our study support the hypothesis that regional signs of small vessel disease may be differentially correlated with executive function, mental processing speed, and memory. Whereas numerous reports from the literature show mixed results with whole-brain SH volumetrics and associations with cognitive functioning, limited reports have demonstrated brain-behavior relationships with hyperintensities in specific brain regions [6,11,40,55-60]. However, one study which examined the effects of focal lesions on verbal fluency found that strategic frontal lobe damage may modulate executive function [61]. More specifically, patients with damage in the medial frontal region, a region which corresponds to the SABRE parcellated MMF region (Figure 2), were shown to perform poorly on the FAS ‘letter-based’ verbal fluency task [62]. Additionally, several studies examining strategic SH on the cholinergic WM tracts have demonstrated correlations with executive, visuospatial, and memory function [63-67]. A novel study (on a group of normal elderly patients) using a combination of structural and functional MRI demonstrated that an increase in SH within the dorsolateral prefrontal cortex was associated with decreases in prefrontal cortex activity during a working memory task [68]. Another study using FLAIR imaging to examine older stroke patients found differential associations with SH, demonstrating that SH volumes in the frontal lobe were associated with cognitive processing speed and attention, while SH volumes in the temporal lobe were associated with memory impairment [9]. Finally, a recent study using a voxel-based analysis method found specific clusters of SH to be associated with executive function and episodic memory [7].

Although the results of our study support this handful of studies reported in the literature, it is unclear why more groups have not reported similar findings, given the widespread availability of imaging techniques which allow SH quantification.

It is likely that the limited number of reports found in the literature is due to methodological differences in quantification of SH as well as a lack of standardized definitions for periventricular, deep white, and lacunar-like, cystic fluid-filled infarcts [2,15,69,70]. Although the increasing popularity of thinner-slice 3D FLAIR images has allowed more automatic segmentation of SH, it has also introduced more variability as FLAIR images have been shown to have lower sensitivity in different brain regions [71]. Various approaches have also been applied for the separation of pvSH from dwSH, where some groups have employed proportional distances from the ventricles to the dura matter [15], whereas other groups employ an arbitrary distance from the ventricles [72]. Our study applied a 3D connectivity algorithm, similar to that of van den Heuvel and colleagues [10] (2006), whose results will clearly vary from the previously mentioned approaches. Additionally, many older studies were based on semi-quantitative data obtained from visual rating scales to estimate SH load [63,73-75] or with the use of volumetric estimates derived by adding slice-by-slice semi-quantitative ratings with spherical shape assumptions for each lesion [76]. Furthermore, a recent meta-study examining differences in lacunar lesion definitions revealed a wide variation in the literature regarding the detection and classification of lacunar lesions and thus recommended a consensus for imaging definitions for small vessel disease [70], now achieved by the STRIVE recommendations [2].

In a recent study examining early- and late-onset AD, a relationship was demonstrated with frontal SH and mental processing speed by using the Trails A score [57]. Unfortunately, cross-comparisons with our study are difficult as this study did not examine the relationship with mental speed using the periventricular and deep white delineations. However, correlations with mental processing speed and SH in the periventricular region have been reported in at least one major longitudinal study, which demonstrated that progression of pvSH was associated with increased time to complete a Stroop test in a large sample of non-demented elderly patients [10]. Although our study was cross-sectional, a similar association was found by using the Trails A trail-making test in a group of demented elderly patients. Interestingly, the volumetric table in the study by van den Heuvel and colleagues [10] reports a mean volume for pvSH at baseline of (4.12 cc), which is comparable to the normal elderly pvSH volume in the current study (4.5 cc).

A possible explanation for this decrease in mental processing speed related to pvSH has been proposed, implicating the long association WM tracts [10,76]. Disruptions from SH in the periventricular region may affect communication between distant multiple cortical brain areas and may result in an overall decrease in speed of processing. In contrast, dwSH may affect communication along the so-called “U-fibers” that connect adjacent areas of the brain and thus are less likely to translate into a significant decrease in processing speed [76]. In this sample, the overall volume of dwSH was small relative to pvSH volumes in both AD and NC (AD: pvSH = 4.5 cc, dwSH = 0.6 cc; NC: pvSH = 1.8 cc, dwSH = 0.3).

This may suggest that the decrease in mental processing speed found in patients with AD may be due in part to some subcortical vasculopathy in the periventricular region related to venous collagenosis [3]. Pathological correlates suggest that pvSH may reflect a form of vasogenic edema resulting from venous insufficiency due to veno-occlusive disease of the deep medullary veins in the periventricular region, which in turn may impair interstitial fluid circulation and exacerbate amyloid angiopathy that is commonly associated with AD [77]. This theory is based on brain pathology imaging work that examined the periventricular venous system in a broad age range (25 to 95 years) of patients at autopsy [78]. The major periventricular and subependymal veins and venules were selectively identified from arteries by using alkaline phosphatase microvascular staining combined with a modified Masson trichrome collagen counterstain. Assessment of this vasculature revealed that the periventricular veins from 65% of subjects over 60 years old had at least 50% stenosis of the periventricular veins because of non-inflammatory collagenous thickening of the venous walls. Moreover, regression analyses revealed that greater venous disease was associated with more severe leukoaraiosis, which was observed as hyperintensities on MRI. Subsequent studies led the authors to conclude that the thickening of the walls of periventricular veins and venules, with collagen subtypes I and III, results in stenosis and occlusion which may restrict venous outflow [79]. This venous collagenosis was believed to be a possible significant contributor to leukoaraiosis, as this venous pathology was likely a progressive and age-related pathology associated with increased WM signal intensity in the periventricular region on MRI of elderly, AD, and vascular dementia patients [80,81].

In our study, no brain-behavior relationships were demonstrated with lacunar lesion subtypes. As stated previously, there is a large variation in detection and identification of lacunes, consensus-driven definitions have yet to be established, and recent controversies in this matter are yet to be resolved [70,82]. For clarification purposes, lacunar volumes in the current study were defined as any hypointense (CSF intensity) voxels on T1 that are found within hyperintense SH-defined voxels on PD/T2. A large number of participants in our sample population had no signs of lacunar infarction as defined by this segmentation method. Although patients with AD in our study had a larger volume of lacunar infarcts compared with NC, it is likely that the wide variation and regional distribution for lacunar lesion volumes in both groups limited correlative power in the analysis.

The results from our study also suggest that poor memory performance is correlated with SH volumes in the left temporal lobe. A recent study report from a Dutch group examining patients with AD (n = 107) found a similar association with hyperintensities in the temporal region and memory by using the Visual Association Test [57]. Another group used a similar region of interest-based method examining SH volumes in a stroke population and found an association with right temporal SH and memory by using a numerical working memory task [9]. In contrast, a composite score was used in our study, which was composed of several memory tests that included a visual reproduction task as well as a verbal memory test. Despite these varied memory components, a correlation was demonstrated with SH volume in the left temporal lobe. Unfortunately, the SABRE VOIs for the temporal lobes were relatively large. Future releases of this software will include further subdivisions of the temporal lobe.

As a final comment, it is important to note that the regional correlations with SH volumes demonstrated in our study were relatively small compared with the large proportion accounted for by atrophy, as indicated by BPF. In large longitudinal studies, such as the Alzheimer’s Disease Neuroimaging Initiative (ADNI), vascular markers have been shown to be associated with brain atrophy. When the Boundary Shift Integral was used as a measure of annualized change in brain volume, a relationship between atrophy and SH was demonstrated in pre-dementia stage participants enrolled in ADNI [83]. However, in the ADNI sample, this relationship was demonstrated in NC participants but not in participants with AD or mild cognitive impairment (MCI). The opposite was demonstrated in our study, which showed SH to be associated with atrophy measures in AD patients but not in NC. This could be due to sample differences and the inclusion of an MCI sample, where a larger AD sample was analyzed in our study (Sunnybrook AD: n = 265 versus ADNI AD: n = 146), and a larger NC and MCI sample in the ADNI study (ADNI NC: n = 197, ADNI MCI: n = 331, versus Sunnybrook NC: n = 100).

Ventricular expansion is another commonly used MRI-based biomarker of atrophy [32]. Longitudinal results from the large Cardiovascular Health Study showed significantly greater vCSF volumes in dementia cases compared with MCI, and MCI compared with NC [33]. Additionally, it was suggested that larger vCSF volumes in NC at baseline may be used to indicate future dementia progression. Future longitudinal analyses examining baseline measures of SH and atrophy may further elucidate the predictive cognitive outcomes of these MRI markers of disease progression.

As our study was cross-sectional, it would be interesting to examine volumetric change over time, particularly with SH subtypes and other brain tissue compartments, to determine whether these changes predict progression patterns in comparison with ADNI data sets. Future directions will examine the inclusion of additional patient groups, including those with MCI, to further examine the relationships between disease progression and mixed pathologies. Diffusion tensor imaging measures of fractional anisotropy and diffusivity and arterial spin labeling perfusion imaging obtained in a subset of these participants will also be added to the correlative modeling.

## Conclusions

In contrast to some previous studies, this study supports the hypothesis that regional signs of small vessel disease, observed on MRI scans of sporadic AD patients and normal elderly individuals, may be differentially associated with specific cognitive functions. These brain-behavior relationships suggest that, while the overarching neurodegeneration and atrophy associated with AD may appear to ‘trump’ subcortical vasculopathies, these subtle, yet measurable, signs of small vessel disease may have potential clinical relevance as targets for modifying vascular risk factors and treatment for this underlying pathology becomes better understood.

## Abbreviations

3D: three-dimensional; AD: Alzheimer’s disease; ADNI: Alzheimer’s Disease Neuroimaging Initiative; BPF: brain parenchymal fraction; CSF: cerebrospinal fluid; dwSH: deep white subcortical hyperintensity; FLAIR: fluid attenuated inversion recovery; FOV: field of view; GM: gray matter; LE: Lesion Explorer; MCI: mild cognitive impairment; MMSE: Mini-mental state exam; MRI: magnetic resonance image; NC: normal control; NEX: number of excitations; PD: proton density; pvSH: periventricular subcortical hyperintensity; SABRE: Semi-Automatic Brain Region Extraction; sCSF: sulcal cerebrospinal fluid; SH: subcortical hyperintensity; STRIVE: STandards for ReportIng Vascular changes on nEuroimaging; ST-TIV: supra-tentorial total intracranial volume; T1: T1-weighted; T2: T2-weighted; TE: echo time; TR: repetition time; vCSF: ventricular cerebrospinal fluid; VOI: volume of interest; WM: white matter.

## Competing interests

The authors declare that they have no competing interests.

## Authors’ contributions

JR participated in the conception and design of the study, drafted the manuscript, performed statistical and imaging analyses, and coordinated all efforts related to the project. AAM performed brain image processing, created neuropsychological composite scores, assisted with statistical analyses, created the tables and figures, and edited the manuscript. CJS provided brain image processing support and assisted in editing the manuscript. DTS provided neuropsychological consultation, was involved in the original design of the large ongoing longitudinal study, and provided critical review for important intellectual content in the manuscript. SEB was the principal investigator; was intimately involved in the conception, design, data acquisition, analysis, interpretation, intellectual content, funding acquisition, manuscript draft, manuscript final edits, manuscript approval for submission, and response to reviewers; was accountable for all aspects of the work, including its accuracy and integrity; and supervised all aspects of the project from start to end. All authors have read and approved the final manuscript.
